# Assessing the Comorbidity Cycle Between Psoriasis and Addiction Based on ICD Coding in the Stockholm Psoriasis Cohort

**DOI:** 10.2340/actadv.v105.41221

**Published:** 2025-01-03

**Authors:** Hannah WECKER, Axel SVEDBOM, Fabio SÁNCHEZ ORREGO, Stefanie ZIEHFREUND, Mona STÅHLE, Alexander ZINK

**Affiliations:** 1 Technical University of Munich, TUM School of Medicine and Health, Department of Dermatology and Allergy, Munich, Germany; 2 Division of Dermatology and Venereology, Department of Medicine Solna, Karolinska Institutet, Stockholm; 3 Dermatology and Venereology Clinic, Karolinska University Hospital, Stockholm; 4 Department of Clinical Neuroscience, Karolinska Institute and Metadonsektionen, Beroendecentrum Stockholm, Stockholm, Sweden

**Keywords:** alcoholism, addiction, cohort studies, comorbidity, psoriasis, substance abuse

## Abstract

The comorbidity cycle between psoriasis and addictions remains unclear. The study aimed to investigate the cumulative incidence of addictions in psoriasis patients and controls in the Stockholm Psoriasis Cohort (SPC). The SPC is an observational cohort study that enrolled psoriasis patients between 2001 and 2005 and matched controls using the Swedish Total Population Register. Data were complemented by medical records from 1987–2013, focusing on 11 addiction diagnoses and the date of their assignment. Overall, 4,545 individuals (56.4% female; median age: 40) were included: 722 psoriasis patients and 3,823 controls. Patients showed 1.4 times (95% confidence interval: 0.98–1.98) higher odds of addiction diagnosis than controls. Alcohol dependency was the most common addiction diagnosis (78.2%), which was more frequent in patients than in controls (94.3% vs 73.6%, *p* = 0.009). Furthermore, patients showed 4.3 times (1.85–11.56) higher odds of receiving an addiction diagnosis after their initial psoriasis diagnosis than before. Results showed a tendency towards a higher risk of addiction in psoriasis patients, suggesting potential psoriasis-triggered addictive behaviour. Nevertheless, both substance abuse triggering psoriasis and chronic psoriasis inflammation triggering addictions have to be considered. In both cases, addictive behaviour needs to be addressed in psoriasis healthcare as a driver for poor disease outcome and comorbidities.

## SIGNIFICANCE

This study investigated the link between psoriasis and addiction, using data from the Stockholm Psoriasis Cohort, including 722 psoriasis patients and 3,823 controls. Psoriasis patients showed 1.4 times higher odds of addiction diagnosis compared with controls, with alcohol dependency being the most common. Patients were 4.3 times more likely to develop an addiction after their psoriasis diagnosis than before. These results showed a tendency towards a higher risk of addiction in psoriasis patients. However, both substance abuse triggering psoriasis and psoriasis triggering addictions have to be considered. Addictive behaviour must be addressed in psoriasis treatment as a cause of poor disease outcomes and comorbidities.

One definition for addiction is the loss of control over drug use or the compulsive seeking and consumption of drugs despite adverse consequences (1, 2). Alcohol, tobacco, opioids, cocaine, and many other legal or illegal substances are consumed in our society (3). Drug addiction involves multiple neural networks including the reward system, the stress system, and the central immune system (4). Confirmed environmental risk factors for addiction include traumatic experiences like physical and sexual abuse, migration, urbanicity, onset of schizophrenia, and cannabis use, smoking, and problematic alcohol use before the age of 18 years (5). Additional risk factors involve perinatal complications, season of birth, early neurotrauma, the number of infections, paternal age (6), and heritability (5, 7). Many diseases are linked to addiction, especially mental disorders (8). However, some studies have observed a link between dermatological diseases and addiction (9, 10). In particular, patients with psoriasis manifest addictive behaviour (compulsory internet use, gambling) and substance-related addictions (alcohol, nicotine, drugs) more often than the general population (11, 12).

Psoriasis is a chronic inflammatory skin disease with a prevalence of 1.92% in Western Europe and 1.50% in the USA and is therefore one of the most frequent dermatological diseases worldwide (13). Recently, substantial progress has been made in developing highly effective therapies based on a deeper understanding of the complex pathogenesis of psoriasis. Moreover, the list of associated comorbidities is expanding. Arthritis, obesity, arterial hypertension, diabetes mellitus, and metabolic syndrome tend to go hand in hand with this skin disease (14, 15). Psoriasis often affects both the body and the mind, and in addition to studying genetics and immunopathogenetic mechanisms, current research has also focused on different personality traits of psoriasis patients and their personal perception of the disease (16, 17). Currently, a clear association between psoriasis and mental health disorders (18), including sleep disorders, depression, anxiety, and suicidality as well as addictive behaviour, is recognized in psoriasis healthcare (11, 19, 20). However, psychiatric disorders may be a consequence of psoriasis as well as a trigger for it (21, 22). Considering recent understandings of psoriasis as a systemic inflammatory disorder beyond the skin, the question arises as to whether addictions are triggered by psoriasis itself or if substance abuse and other addictions are triggers for psoriasis.

To start teasing out that relationship, the study aimed at investigating the temporal connection between the onset of psoriasis and the onset of addiction, as well as describing the cumulative incidence of addiction to different substances among psoriasis patients and controls from the Stockholm Psoriasis Cohort.

## MATERIALS AND METHODS

### Study design

The Stockholm Psoriasis Cohort (SPC) is a non-interventional observational cohort study that enrolled patients with psoriasis within 12 months of disease onset and matched controls between 2001 and 2005 predominantly in Stockholm, Sweden. All participants provided written informed consent prior to study inclusion. The study was reviewed and approved by the Regional Committee of Ethics and followed the Strengthening the Reporting of Observational Studies in Epidemiology (STROBE) reporting guideline for cohort studies. Detailed information on SPC and data collection have been described elsewhere (23, 24).

### Participants

Patients aged 15 years or older with their first onset of psoriasis lesions on non-hairy skin within the past 12 months were eligible to participate in the study. Using the Swedish Total Population Register, 6 matched controls were identified based on age, sex, and postal code to optimize socioeconomic matching for each patient with psoriasis. In total, the SPC population comprised 4,709 participants with 753 patients and 3,828 controls (23, 24).

### Data sources/measurement

SPC data were enhanced by data from questionnaires and medical records from the National Patient Register (NPR) (25).

The NPR was established in 1964 and has complete coverage of inpatients in Sweden since 1987. More than 99% of all somatic and psychiatric discharges are registered in the NPR. Since 2001, regions have been obliged to report specialist outpatient physician visits except those for primary care, albeit there were regional differences in data quality in the first years. Variables in the register include date and length of stay, diagnoses, surgical procedures, and Diagnosis Related Group (DRG) codes (25).

Due to the complete coverage in inpatient care since 1987, only the period from 1987 to 2013 was considered in this study. We therefore examined 2 time periods: 1987–2013, including all primary diagnoses within this period, and 2001–2013 with all corresponding primary diagnoses after the SPC enrolment. Psoriasis diagnosis was excluded from the medical records. The exact date of each diagnosis was recorded and categorized as before/after individual enrolment date, for both patients and controls. The date of study enrolment, which is considered to be the date of psoriasis diagnosis for each patient, was the same for psoriasis patients and their respective controls. Individuals diagnosed with an addiction both before and after SPC enrolment were grouped as “before+after”. An addiction diagnosis was defined by the following diagnostic codes according to the tenth revision of the International Statistical Classification of Diseases and Related Health Problems (ICD-10): alcohol-related disorders (F10), opioid-related disorders (F11), cannabis-related disorders (F12), sedative, hypnotic, or anxiolytic-related disorders (F13), cocaine-related disorders (F14), other stimulant-related disorders (F15), hallucinogen-related disorders (F16), nicotine dependence (F17), inhalant-related disorders (F18), other psychoactive substance-related disorders (F19), or abuse of non-psychoactive substances (F55). Addiction diagnoses coded in ICD-9 were decoded into ICD-10 using tables provided online by the Swedish National Board of Health and Welfare (https://www.socialstyrelsen.se/).

### Sample size

Due to missing data at enrolment, 31 patients were excluded. Evidence of psoriasis in 133 controls led to the exclusion of these participants from the present analysis. Therefore, a total of 4545 subjects were included in the analysis, consisting of 722 patients and 3,823 controls. In this study, we have considered the controls as a cohort for comparison rather than as matched controls, to maximize the sample size.

### Statistical methods

The study analysed participants individually in inpatient, outpatient, and total settings. Individuals diagnosed in both inpatient and outpatient settings were included only once in the total category. Cumulative incidences of addictions overall and for different addiction diagnoses were calculated. Furthermore, the percentage of participants who were diagnosed with addiction before, after, or before and after study enrolment was also evaluated. Odds ratio (OR) and 95% Wald confidence interval (CI) were calculated based on cross-tables. Differences between patients and controls were tested with the χ^2^ test and Fisher’s exact test (for small sample sizes) for categorical variables and with the two-sample *t*-test for continuous variables. To test for differences in the proportions of participants with an addiction diagnosis before and after study entry, an exact McNemar test was used separately for patients, controls, and total. For this test, participants diagnosed before and after study entry (“before+after”) were counted for both “before” and “after”. All *p*-values reported are two-sided and significance level was set to 0.05. A missing diagnosis date was considered as no diagnosis, which concerned 34 controls. Analyses were implemented in R, version 4.0.4 (R Foundation for Statistical Computing, Vienna, Austria).

## RESULTS

### Descriptive data

A total of *n* = 4,545 individuals participated in this study, with 722 being patients and 3,823 being part of the control group (**Table I**). More than half of the participants (56%, *n* = 2,550) were female, and the median age was 40 years (interquartile range [IQR]: 28–56). Overall and in the outpatient setting, psoriasis patients received significantly more primary diagnoses than controls in both time periods (*p* < 0.001), while the number of diagnoses in inpatients was approximately the same (2 diagnoses, *p* = 0.347).

**Table I:** Demographics and number of addiction diagnoses for psoriasis patients, controls, and overall Stockholm Psoriasis Cohort (SPC) participants

Variable	Overall	Patients	Controls	*p*-value	OR^a^ [95% CI]
Sample size, *n* (%)	4,545	722 (15.9)	3,823 (84.1)		
Female sex, *n* (%)	2,550 (56.4)	407 (56.4)	2143 (56.4)		
Age, median (IQR)	40 (28–56)	40 (27–55)	40 (28–57)		
Diagnoses per subject, median (IQR)					
Total	7 (4–12)	8 (5–15)	7 (3–12)	< 0.001	
Inpatients	2 (1–4)	2 (1–4)	2 (1–4)	0.347	
Outpatients	6 (3–10)	7 (4–12)	5 (3–10)	< 0.001	
Diagnoses per subject after SPC enrolment, median (IQR)					
Total	5 (3–10)	6 (3–11)	5 (3–9)	< 0.001	
Inpatients	2 (1–3)	2 (1–3)	2 (1–3)	0.666	
Outpatients	5 (2–9)	6 (3–10)	5 (2–8)	< 0.001	
Subjects with addiction diagnosis, *n* (%)					
Total	204 (4.5)	42 (5.8)	162 (4.2)	0.060	1.4 [0.984, 1.979]
Inpatients	128 (4.5)	16 (2.2)	112 (2.9)	0.288	0.8 [0.442, 1.276]
Outpatients	147 (3.2)	34 (4.7)	113 (3)	0.015	1.6 [1.096, 2.401]
Subjects with addiction diagnosis after SPC enrolment, *n* (%)					
Total	156 (3.4)	35 (4.8)	121 (3.2)	0.023	1.6 [1.061, 2.290]
Inpatients	77 (1.7)	10 (1.4)	67 (1.8)	0.483	0.8 [0.403, 1.538]
Outpatients	134 (2.9)	32 (4.4)	102 (2.7)	0.010	1.7 [1.128, 2.537]
Addiction diagnoses per subject, median (IQR)	2 (1–6)	2 (1–6)	2 (1–6)	0.259	
Addiction diagnoses per subject after SPC enrolment, median (IQR)	3 (1–8)	3 (1–7)	3 (1–9)	0.166	

a Odds ratio (OR) calculated for patients and controls (controls as reference).

*n*: number; IQR: interquartile range; CI: confidence interval.

### Addiction diagnosis

The total number of persons with an addiction diagnosis between 1987 and 2013 was 204, with patients (5.8%) being slightly more likely to be affected than controls (4.2%, *p* = 0.060, Table I). The odds of an addiction diagnosis were 1.4 (95% CI: 0.984, 1.979) times higher in patients than in controls. When examining the relationship between addiction diagnosis and psoriasis severity (measured by the Psoriasis Area and Severity Index [PASI]), no significant differences in baseline PASI scores were found between psoriasis patients with (median: 3.55 [IQR: 2.175, 5.775]) and without an addiction diagnosis (3.10 [1.675, 5.100]; *p* = 0.309).

*After SPC enrolment.* Psoriasis patients (4.8%) were more likely to have a primary diagnosis of addiction than controls (3.2%, *p* = 0.023) after their SPC enrolment. Therefore, the risk of an addiction diagnosis was 1.6 (95% CI: 1.061, 2.290) times higher in patients than in controls, which was also the case in the outpatient setting (OR 1.7 [95% CI: 1.128, 2.537]). Patients had a numerically lower risk for addiction than controls with respect to inpatient settings (OR 0.8 [95% CI: 0.403, 1.538]).

**Fig. 1:**
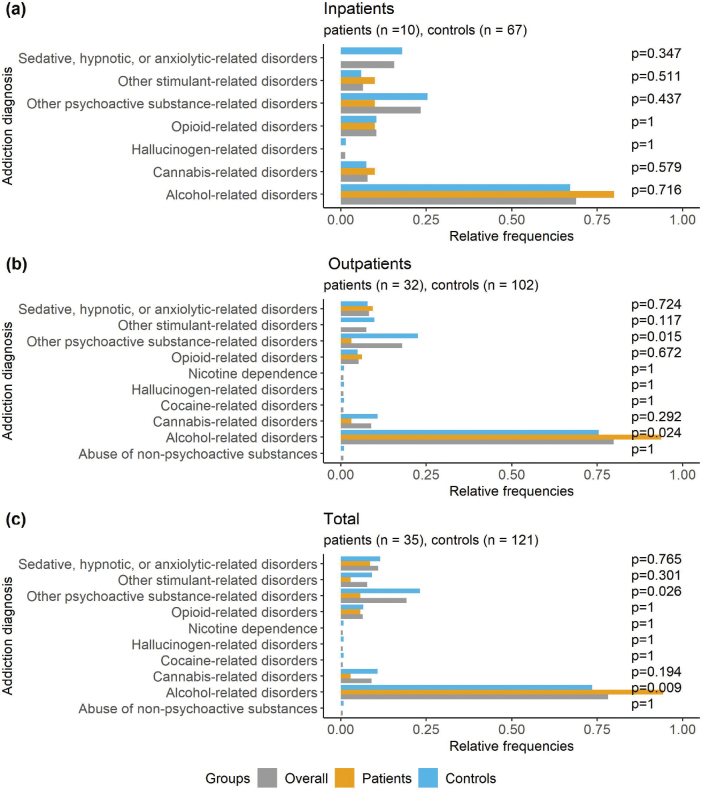
**Frequencies of different addiction diagnoses after Stockholm Psoriasis Cohort (SPC) enrolment in (A) inpatients, (B) outpatients, and (C) total for psoriasis patients, controls, and overall participants of the SPC.** *P*-values were reported for *χ*^2^ tests or Fisher’s exact tests (for small sample sizes) to assess differences between psoriasis patients and controls.

*Types of addiction diagnosis after SPC enrolment.* Overall, the most frequent addiction diagnoses among addicted study participants (*n* = 156) were alcohol-related disorders (78.2%), other psychoactive substance-related disorders (19.2%), sedative, hypnotic, or anxiolytic-related disorders (10.9%), and cannabis-related disorders (9.0%). Among participants diagnosed with addiction, alcohol-related disorders were more common in patients with psoriasis than in controls (94.3% vs 73.6%, *p* = 0.009), whereas the opposite was observed for psychoactive substance abuse: 5.7% of patients and 23.1% of controls (*p* = 0.026, **Fig. 1**C). Similar results were obtained in outpatient (Fig. 1B) and inpatient settings (Fig. 1A). However, opioid-related disorders (10.4%) were diagnosed more frequently than cannabis-related disorders among inpatients (7.7%, Fig. 1A).

### Onset of addiction

Among participants diagnosed with an addiction between 1987 and 2013 (*n* = 204), the odds for an addiction diagnosis after study commencement were about 2.4 times (95% CI 1.681, 3.401) higher than before (*p* < 0.001, **Table II**). Seven patients (16.7%) were diagnosed with an addiction before their psoriasis diagnosis, 30 (71.4%) afterwards, and 5 (11.9%) both before and after. Therefore, receiving an addiction diagnosis after psoriasis diagnosis was 4.3 times (95% CI: 1.845, 11.559) more likely to happen than before (*p* < 0.001). In controls, the risk of an addiction diagnosis was 2.0 times (95% CI: 1.394, 3.053) higher after the start of the study (*p* < 0.001).

**Table II:** Onset of addiction diagnosis before and after Stockholm Psoriasis Cohort (SPC) enrolment in inpatients, outpatients, and total for psoriasis patients, controls, and overall participants

Factor	Sample size	Time of addiction diagnosis, *n* (%)			*p*-value^a^	Before vs after	
		Before	After	Before+after		OR^b^ [95% CI]	*p*-value^c^
Total	204	48 (23.5)	114 (55.9)	42 (20.6)		2.4 [1.681, 3.401]	< 0.001
Patients	42	7 (16.7)	30 (71.4)	5 (11.9)	0.077	4.3 [1.845, 11.559]	< 0.001
Controls	162	41 (25.3)	84 (51.9)	37 (22.8)		2.0 [1.394, 3.053]	< 0.001
Inpatients	128	51 (39.8)	51 (39.8)	26 (20.3)		1.0 [0.665, 1.504]	1
Patients	16	6 (37.5)	6 (37.5)	4 (25)	0.884	1.0 [0.267, 3.741]	1
Controls	112	45 (40.2)	45 (40.2)	22 (19.6)		1.0 [0.647, 1.546]	1
Outpatients	147	13 (8.8)	120 (81.6)	14 (9.5)		9.2 [5.198, 17.840]	< 0.001
Patients	34	2 (5.9)	29 (85.3)	3 (8.8)	0.930	14.5 [3.668, 125.407]	< 0.001
Controls	113	11 (9.7)	91 (80.5)	11 (9.7)		8.3 [4.412, 17.156]	< 0.001

a *p*-value of the comparisons between patients and controls tested with Fisher’s exact test.

b Odds ratio (OR) calculated for before and after study entry with “before+after” counted for both “before” and “after” (before as reference).

c *p*-value of the comparison between before and after study entry tested with McNemar’s test.

*n*: number; IQR: interquartile range; CI: confidence interval.

In 147 outpatients, the risk of an addiction diagnosis was 9.2 times (95% CI: 5.198, 17.840) higher after study entry than before (*p* < 0.001). High ORs were also observed for psoriasis patients (OR: 14.5 [95% CI: 3.668, 125.407], *p* < 0.001) and controls (OR: 8.3 [95% CI: 4.412, 17.156], *p* < 0.001) in the outpatient setting.

However, among 128 inpatients, the risk of an addiction diagnosis after study entry was the same as the risk before (OR: 1.0 [95% CI: 0.665, 1.504], *p* = 1.000). Similar results were obtained for psoriasis patients (OR: 1.0 [95% CI: 0.267, 3.741], *p* = 1.000) and controls (OR: 1.0 [95% CI: 0.647, 1.546], *p* = 1.000) in the inpatient setting.

No significant dependence between time of diagnosis and status as a patient or control was observed for inpatients, outpatients, and total.

## DISCUSSION

This study assesses the relationship between psoriasis and addictive disorders in a large cohort of psoriasis patients and controls in Sweden. A higher cumulative incidence of addiction was found for psoriasis patients compared with controls, particularly alcohol-related disorders. In addition, psoriasis patients had a higher risk of receiving an addiction diagnosis after being diagnosed with psoriasis compared with controls in the same time period. However, no conclusive evidence was found for an overall higher risk of addiction in psoriasis patients.

Psoriasis can negatively impact the quality of life in different ways, such as interfering with social interactions due to stigmatization, social inhibition, and embarrassment, which consequently causes greater susceptibility to anxiety and depression (26). In turn, a greater susceptibility to addictive behaviours might be expected, which also concerns individuals with psoriasis (27). A negative impact on mental health is related to elevated use and abuse of substances including alcohol, tobacco, cannabis, and other drugs that are commonly found to be comorbid with psychiatric conditions in adolescents (28). Another explanation may be that pro-inflammatory cytokines associated with psoriasis are linked to coexistent mental health disorders and addictions (29). However, there was no association between psoriasis severity and addictive behaviour observed in this study.

In line with the literature, we found alcohol abuse to be the main addiction in patients with psoriasis (30). Previous studies have also demonstrated that psoriasis patients consume significantly more alcohol than healthy controls (31) with a 2 to 3 times higher alcohol intake (32–34) and a higher rate of excessive drinking (11, 35). Previous studies focusing on addiction disorder in psoriasis patients also reported excess rates for daily smoking (17.1% vs 3.1%) (12, 36), which was not seen in this study. However, this difference in results may be explained by our method of using the ICD-10 codes for nicotine dependence instead of participant self-assessment data for daily smoking as seen in another study (12).

Regarding the abuse of sedative, hypnotic, anxiolytic, and opioid-related disorders, there were no differences observed between psoriasis patients and controls. Surprisingly, other psychoactive substance-related disorders (F19) were more common in controls than in patients. Available cross-sectional studies screening for illicit drug use described psoriasis patients as being at a higher risk for heroin, cocaine, methamphetamine, and cannabis abuse compared with non-psoriasis patients (37). However, the evaluation of drug use was based on self-assessment questionnaires concerning drug use, whereas our study was based on ICD-10 codes, which may underestimate the real rate of illicit drug use in psoriasis.

Overall, addiction diagnoses were significantly more frequent after psoriasis onset, which has not been reported by other studies so far. One reason may be that, after the initial psoriasis diagnosis, patients have increased contact with healthcare professionals and are therefore more likely to be diagnosed with other conditions including addictions. Additionally, chronic diseases lead to unexpected life changes. For example, patients who are newly diagnosed with cancer are at higher risk of mental disorders, even in patients with no previous history of mental disorders (38). Recent studies showed that patients with untreated psoriasis have as much impairment of psychological well-being as patients with cancer or other major medical diseases (39). Moreover, a study on children and teenagers with psoriasis reported that these young patients were significantly more at risk of developing psychiatric disorders, especially depression and anxiety, compared with members of a healthy control group (40). In interpreting this finding, it should be noted that this result was found in a limited number of cases (7 vs 35 psoriasis patients).

The consumption of alcohol can exacerbate existing psoriasis and may also lead to a less favourable treatment response and non-adherence (27, 41–43). Moreover, psychological factors (e.g., psychological distress) may result in a suboptimal treatment response in psoriasis (44). Consequently, there appears to be an endless cycle between disease exacerbation, decrease in mental health, and addiction. Weekly alcohol consumption, verified by measuring the alcohol marker phosphatidylethanol (Peth) in whole blood, was shown to correlate with disease severity in psoriasis (45, 46). One influential extrinsic factor that affects and triggers psoriasis is psychological stress (47). Chronic stress may not only lead to exacerbation of the disease but also to the development of different mental disorders like depression and anxiety (19). In summary, the consequences of a newly diagnosed, stigmatizing skin disease like psoriasis can lead to chronic stress, impaired quality of life, and eventually psychological comorbidities, potentially resulting in a vicious cycle that precipitates substance abuse (27).

Conversely, habitual drinking and light to moderate alcohol intake is known to be associated with an anti-inflammatory effect and a decreased risk for many cardiovascular diseases, diabetes mellitus, congestive heart failure, stroke (48), and depression (49). Low-dose alcohol intake could therefore also have a protective effect in psoriasis development, but further research is needed to investigate whether a specific threshold of alcohol can induce or worsen psoriasis (50).

Across the entire study period, a tendency towards a higher risk of addiction was observed in psoriasis patients compared with healthy controls, although this result was not statistically significant. However, a statistically significant higher risk of addiction was shown for psoriasis patients in the period after SPC enrolment.

While the results lack conclusive evidence, they suggest an increased risk in psoriasis patients and further studies are needed to continue investigating the comorbidity cycle between psoriasis and addiction.

### Limitations

Our study is the first to describe alcohol consumption and dependency based on physician-made diagnoses documented with ICD codes. However, this means that no detailed comparisons with other publications could be made. Using ICD codes can also underestimate addiction in patients, as less conspicuous addictive behaviour like moderate drinking can be concealed by participants. The absence of more recent medical data constitutes a limitation, as it may have influenced potential outcomes differently. Primary outpatient diagnoses were only available between 2001 and 2013, which consequently led to a lower number of diagnoses prior to study enrolment and therefore clearer results when examining the onset of addiction. Furthermore, as in any other disease registries, missing or incomplete data, false coding, and less supervised registry enrolment compared with randomized trials may represent a source of bias.

### Conclusion

Psoriasis may drive addictions, but it is also triggered and worsened by active substance abuse. As addictions and especially alcoholism seemed to be more common in psoriasis patients compared with controls, the awareness of this relationship among treating physicians is essential. Psychological and addictive comorbidities lead to the worsening of the disease and poor treatment outcome and require special attention in individual patient care. Substance abuse often begins during the developmental period of psoriasis and early detection and intervention could prevent serious consequences. Understanding psoriasis as a systemic disease is required for multidisciplinary strategies to identify and manage the affected population. The influence of addictive behaviour and related needs in psoriasis healthcare should be investigated in further studies to establish a “sustained psoriasis treatment”.
